# A rare case of recurrent epidermoid anal cancer treated with cytoreductive surgery and hyperthermic intraperitoneal chemotherapy—case report

**DOI:** 10.1186/s12957-020-01935-3

**Published:** 2020-07-04

**Authors:** Vera Pravong, Alexandre Brind’Amour, Lucas Sidéris, Pierre Dubé, Jean-François Tremblay

**Affiliations:** 1 Department of General Surgery, University of Montreal, Montreal, Quebec, Canada; 2 The Centre de Recherche de l’Hôpital Maisonneuve-Rosemont, Université de Montréal, 5415 boulevard de l’Assomption, Montreal, QC H1T 2M4 Canada; 3 Division of Surgical Oncology, Maisonneuve-Rosemont Hospital, Montreal, Quebec Canada; 4 Division of Colorectal Surgery, Maisonneuve-Rosemont Hospital, Montreal, Quebec Canada

**Keywords:** Carcinomatosis, Peritoneal metastases, Epidermoid, Anal cancer, Recurrent, HIPEC, CRS

## Abstract

**Background:**

Anal cancer is a rare cancer with chemoradiation being the mainstay of treatment for locoregional presentation. In North America, the most common subtype is anal squamous cell carcinoma (epidermoid). A surgical approach is considered for persistent or recurrent anal disease and systemic chemotherapy for metastatic disease. We are presenting a unique case of recurrent anal cancer with isolated peritoneal malignancy, an oligometastatic state which is rare in itself. It was treated with cytoreductive surgery and hyperthermic intraperitoneal chemotherapy. There are currently no clear guidelines for the aforementioned presentation. The discussion drew on the feasibility and safety of this approach.

**Case presentation:**

A 68-year-old woman diagnosed with an epidermoid anal cancer (stage 3B) was initially treated with chemoradiation therapy (Standard Nigro Protocol) in 2014. At the 5-year mark post-treatment, she was diagnosed with a recurrent anal epidermoid cancer in the form of isolated peritoneal carcinomatosis proven by biopsy. After declining systemic chemotherapy, she underwent cytoreductive surgery and hyperthermic intraperitoneal chemotherapy with Mitomycin-C©. Peritoneal carcinomatosis index was evaluated at 10, and intraoperative frozen sections were positive for carcinoma of epidermoid origin compatible with anal cancer. A completeness of cytoreduction score of 0 was achieved during the cytoreductive surgery, and her hospital course was unremarkable. She remains disease-free 12 months later.

**Conclusions:**

To our knowledge, this is the first case reporting the disease presentation of anal cancer with oligometastatic dissemination to the peritoneum. Cytoreductive surgery and hyperthermic intraperitoneal chemotherapy were performed. Thus far, this approach seems to be a safe and feasible option for short-term control of the disease.

## Introduction

Anal cancer (AC) is a rare malignancy which accounts for 0.5% of all new cancer cases and represents 2.7% of all gastrointestinal (GI) cancers [1, 2]. In North America, the most common subtype is anal squamous cell carcinoma (SCC-epidermoid) [1]. The incidence of AC is currently rising, in both men and women, due to increasing rates of human papilloma virus infection [3]. Early-stage localized AC is managed with combined chemoradiation therapy (CRT), and this sphincter-preserving approach is considered standard of care [4]. The salvage abdominoperineal resection (APR) surgery is reserved for cases of persistent anal disease after failure to respond to initial treatment or for recurrent disease [5]. Metastatic disease is usually treated with systemic chemotherapy [6]. The overall 5-year survival (OS) rate of AC is about 68% [1, 2]. We herein present the case of a patient treated with cytoreductive surgery (CRS) and hyperthermic intraperitoneal chemotherapy (HIPEC) for recurrent epidermoid AC with isolated peritoneal metastases.

## Case presentation

**Fig. 1:**
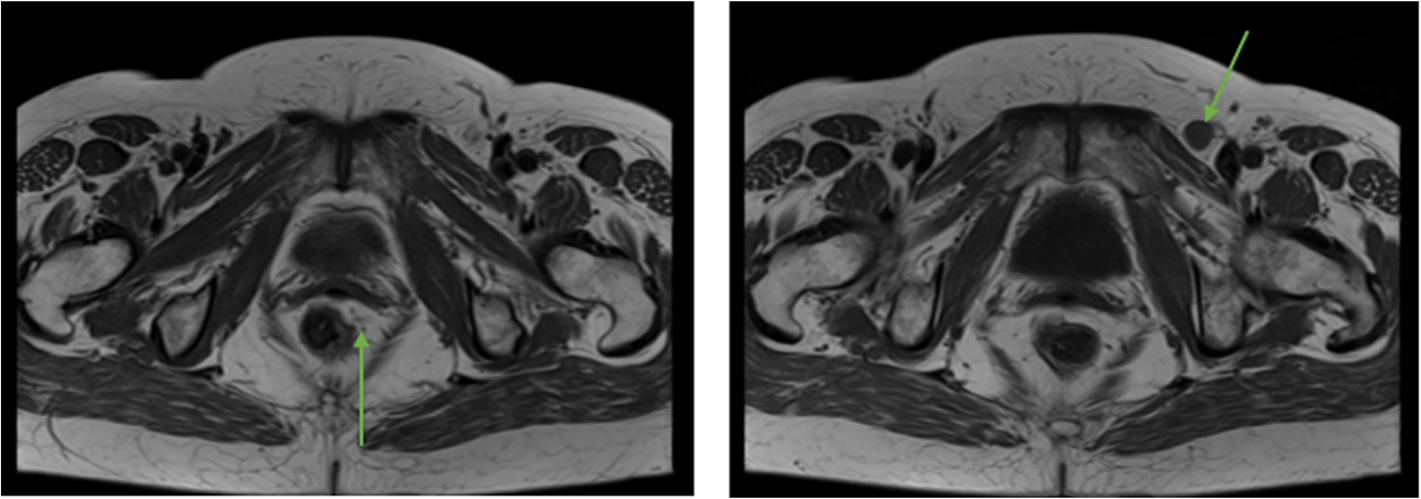
MRI axial images showcasing the initial 2014 tumor at the anorectal junction on the left lateral border (6 × 6 mm) and the left inguinal lymph node (1.2 × 1.2 cm)

A 68-year-old woman was diagnosed with poorly differentiated, human papillomavirus positive, basaloid SCC (T2N2, stage IIIB) and treated with CRT (Standard Nigro Protocol including infusional 5-fluorouracil, Mitomycin-C©, and 30 Gy of radiation) in 2014 at another healthcare institution. The tumor was located at the proximal anal canal, without extension to the external anal sphincter or intersphincteric fat, and it did not reach the peritoneal reflection (Fig. 1). The response to initial treatment was complete, and the course of disease after treatment was unremarkable.

In August 2018, she was referred to our institution for evaluation of a possible AC recurrence. At her initial visit, she reported constipation, transient anal bleeding, dysuria, and pollakiuria. The physical examination was unremarkable, and anoscopy was also normal. A positron emission tomography (PET) scan done 4 months earlier at the referring institution revealed multiple hypermetabolic pelvic peritoneal nodules. The largest and most intense nodule was anterior to the left common iliac artery measuring 1.8 cm. An abdominal-pelvic computed tomography (CT) also described these prominent pelvic implants, the first and largest at the left common iliac chain measuring 2.6 × 2 cm, with a smaller second satellite lesion (1 cm) located inferiorly. The third implant lay anterior to the mid-sigmoid colon and posterior to the uterus (2.5 × 1.6 cm). The fourth implant was located lateral to the uterus and medial to the sigmoid colon (0.8 cm).

**Fig. 2:**
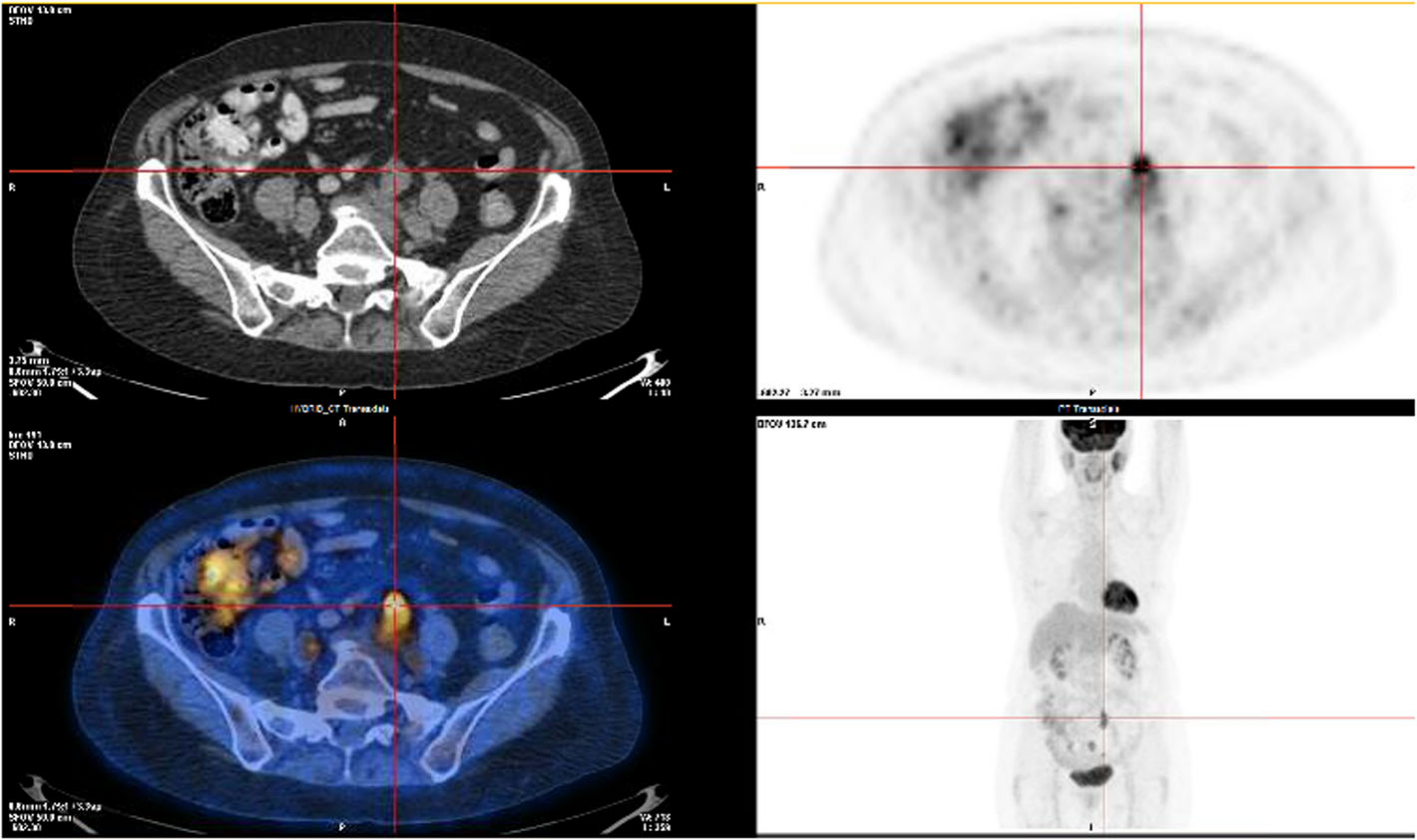
PET-CT axial and coronal images showcasing the hypermetabolic peritoneal carcinomatosis implants in the abdomino-pelvic region

**Fig. 3:**
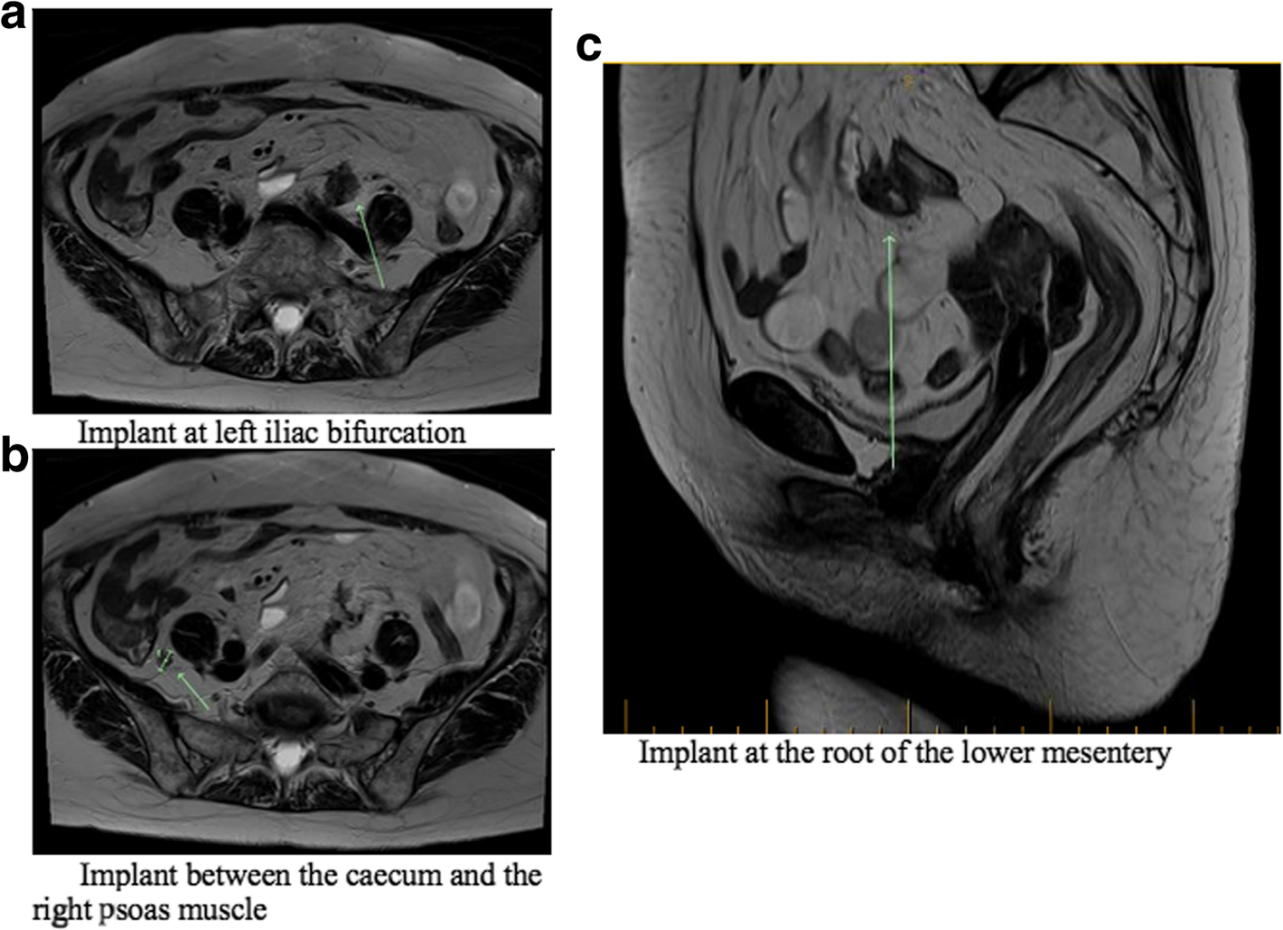
MRI axial and sagittal images showcasing the peritoneal carcinomatosis implants in the abdomino-pelvic region

In light of these results, a full diagnostic work-up was undertaken to investigate the probable recurrence of her AC. At this point, the patient had not received any local or systemic treatment before being referred to our group, and no tissue biopsy had been obtained. A new PET scan study done in our institution reported a mild progression of the nodules, with a dominant implant still along the left common iliac chain (Fig. 2). There was no sign of local recurrence nor any signs of hepatic, adrenal, or bone metastasis. As seen in Fig. 3, the magnetic resonance imaging (MRI) study supported the PET scan findings and revealed at least three distinctive sites of metastatic implants with no evidence of anal or pelvic lymph node recurrence. One was located in front of the left iliac bifurcation (2.5 × 2 cm), another implant was noted at the root of the lower mesentery (1.3 × 1 cm), and there was also an implant between the caecum and the right psoas muscle (1 × 1 cm).

A diagnostic laparoscopy, colonoscopy, and complete anal and gynecological examination were performed under general anesthesia as part of the work-up. The anal canal and the inferior portion of the rectum were normal. A formal vaginal examination was impossible because of a stenosis secondary to prior radiation therapy, but the accessible vaginal mucosa was soft and normal. During the colonoscopy, biopsies were taken in the anal canal and were all found to be negative. Biopsies of peritoneal implants (parasigmoid and left pelvic) confirmed the diagnosis of metastatic epidermoid AC with basaloid traits. Moreover, immunochemistry studies were positive for CK5/6 and P40 markers, hence reinforcing the proposed diagnosis of recurrence of her initial 2014 epidermoid AC.

The patient’s chart was subsequently discussed at the institution’s tumor board and among international experts. The patient hesitated for several months to undergo surgery or to have systemic treatment. A PET scan in early November 2018 demonstrated a slight progression of peritoneal carcinomatosis (PC) with no other lesions reported. Management options were discussed with the patient and her family. After declining systemic chemotherapy, she agreed to undergo CRS with HIPEC to address her recurrent AC.

The patient underwent surgery 1 week later. A midline laparotomy was performed, and the peritoneal cavity was thoroughly inspected for all sites of metastatic disease. The peritoneal carcinomatosis index (PCI) was evaluated at 10. The patient underwent complete CRS including an omentectomy, ileocecal resection, anterior resection of the recto-sigmoid colon to address nodules on the mesentery, and on the wall of the sigmoid colon, as well as a total abdominal hysterectomy and bilateral salpingo-oophorectomy. The remaining nodular implants were also removed at the angle of Treitz, on the small bowel mesentery, on the right retroperitoneum, and in the pelvis. Intraoperative frozen sections were positive for epidermoid carcinoma, hence compatible with the initial AC. A completeness of cytoreduction score (CC) of 0 was achieved during the CRS (disease completely resected). After completion of intestinal continuity, a closed-technique HIPEC was performed with Mitomycin-C© (fixed 20 mg) at 42 °C for 90 min. The surgery was well tolerated, and the patient was transferred to the post-anesthesia care unit in a stable condition. Her post-operative course was unremarkable, and she was discharged on post-operative day 17. The final pathology report of the peritoneal implants was positive for poorly differentiated basaloid SCC. Since the patient had already refused systemic chemotherapy preoperatively and was now considered disease-free, she was not given adjuvant therapy. She was followed at the outpatient clinic every 4 months with a physical exam and alternating CT scans and PET scans. At 12 months, she remains disease-free, with no sign of recurrence at the last PET scan study.

## Discussion

Anal cancer is a rare condition, and CRT is the mainstay treatment for locoregional disease. Stage I/II ACs have a 5-year relative survival rate of 80% [7]. A study by Dewdney et al. reported that 20% of treated AC cases will progress and develop distant metastasis [6]. The 5-year relative survival rate for stage IV disease varies from 10 to 31% [7]. Commonly, AC metastasizes to the liver and lungs [6, 8]. Metastatic and recurrent AC are challenging conditions to treat with management options often limited to systemic chemotherapy [4], and the median survival rates for cases with distant metastases ranges from 8 to 34 months [6]. Clinical trials assessing the use of radiosensitizers and immune checkpoint inhibitors as new therapies for advanced AC are currently ongoing [9–11].

Oligometastatic disease is a particular presentation of anal SCC, and recent literature has demonstrated that organ-directed therapies in carefully selected patients may offer long-term disease control [12, 13]. A multidisciplinary approach, including surgery, should be considered for eligible patients. For example, Pawlik et al. [8] reported a median OS of 22.3 months for a series of patients that underwent hepatic resection of metastatic SCC. Even though most of the patients in the study had a recurrence, long-term survival was achievable for patients with limited disease and complete R0 resection. Slafani et al. [14] reported a median overall survival of 31 months for patients with liver or pulmonary resection of metastatic SCC, with a few patients still alive and disease-free at 5 years. In this current case, the patient was initially offered systemic chemotherapy alone, known to be the mainstay treatment for metastatic anal SCC [15], but she refused it. Since her disease was considered oligometastatic to the peritoneum only, she was offered the surgical option.

To our knowledge, this is the first case reported in the literature of recurrent epidermoid AC with PC treated with CRS and HIPEC. CRS ± HIPEC is a standard treatment for primary peritoneal surface malignancy (PSM), as well as PSM arising from colorectal and appendicular cancers, including pseudomyxoma peritonei [16–19]. This approach can also be considered for PSM from ovarian or gastric carcinomas [20, 21]. A few retrospective studies have been published in recent years relating the international experience of treating patients with complete CRS and HIPEC for other, unusual cancers [22–24]. Although these studies did not include AC patients, the consensus was that for carefully selected patients with rare cancer sites of origin, isolated metastases to the peritoneum and reasonable PCI, there was a potential survival benefit to proceed with CRS and HIPEC. In this case, the patient presented with a metachronous disease, low to intermediate PCI, no evidence of locoregional recurrence or extraperitoneal disease, and a safe and feasible CC-0 CRS. This allowed for consideration of an aggressive but potentially curative approach for this patient. The choice of using Mitomycin-C© as HIPEC regimen was made based on its known efficiency in the treatment of anal SCC (as part of the Standard Nigro protocol).

The exact mechanism for peritoneal dissemination of anal SCC is unknown. Peritoneal metastases usually develop from intra-peritoneal cancer origins, which are almost always adenocarcinomas. A few cases of SCC from other sites (esophagus, ovaries, head, and neck) have been reported in the literature, without clear explanation for such an uncommon metastatic pattern [25]. Advanced lymph node metastases, advanced primary lesion, and absence of local control have been suggested as potential risk factors. Previous surgical field contamination with tumor deposits has also been suggested. In this case, the patient had not been operated on before, and there was no tumor extension to the peritoneal cavity, but there was advanced lymph node extension at initial presentation. Furthermore, the patient presented with basaloid SCC, a histologic subtype associated with increased risk of local and distant recurrence [26]. While it does not explain why the recurrence site was the peritoneal cavity, this patient certainly had risk factors for recurrence.

## Conclusion

Anal cancer with oligometastatic dissemination to the peritoneum is a very rare disease presentation. Although this is the first case reporting such an approach for this etiology, CRS and HIPEC seem to be a safe and feasible option, at least for short-term control of the disease.

## Data Availability

Data sharing is not applicable to this article as no datasets were generated or analyzed during the current study.
